# Association of CD209 (DC-SIGN) rs735240 SNV with paucibacillary leprosy in the Brazilian population and its functional effects

**DOI:** 10.1590/0074-02760220014

**Published:** 2022-06-10

**Authors:** Giovanna Valle Germano, André Flores Braga, Rodrigo Mendes de Camargo, Priscila Betoni Ballalai, Ohanna Cavalcanti Bezerra, Fernanda Saloum de Neves Manta, Andréa de Faria Fernandes Belone, Cleverson Teixeira Soares, Pranab Kumar Das, Milton Ozório Moraes, Ana Carla Pereira Latini, Vânia Niéto Brito de Souza

**Affiliations:** 1 Faculdade de Medicina de Botucatu, Programa de Pós-Graduação em Doenças Tropicais, Botucatu, SP, Brasil; 2 Secretaria de Estado da Saúde de São Paulo, Instituto Lauro de Souza Lima, Bauru, SP, Brasil; 3 Fundação Oswaldo Cruz-Fiocruz, Instituto Oswaldo Cruz, Laboratório de Hanseníase, Rio de Janeiro, RJ, Brasil; 4 University of Birmingham, College of Medical and Dental Sciences, Division of Infection and Immunity, Department of Clinical Immunology, Edgbaston, UK

**Keywords:** DC-SIGN, Mycobacterium leprae, leprosy, single nucleotide polymorphism

## Abstract

**BACKGROUND:**

Leprosy, caused by *Mycobacterium leprae*, is a public health problem in Brazil that affects peripheral nerves, resulting in physical disabilities. During host-pathogen interactions, the immune response determines leprosy outcomes from a localised (paucibacillary) form to a disseminated (multibacillary) form. The recognition of *M. leprae* involves the DC-SIGN receptor, which is present on the dendritic cells (DCs) and participates in immune activation.

**OBJECTIVES:**

To evaluate the association of polymorphisms in the promoter region of the gene encoding DC-SIGN (*CD209*) and the clinical form of leprosy, and to investigate its functional effects.

**METHODS:**

The study population included 406 leprosy patients from an endemic area in Brazil [310 multibacillary (MB); 96 paucibacillary (PB)]. A functional evaluation based on the effects of the single nucleotide variant (SNV) associated with PB leprosy on the specific immune response was also performed.

**RESULTS:**

The GA genotype and the presence of the A allele of rs735240 (-939G>A) were associated with PB leprosy [OR: 2.09 (1.18-3.69) and 1.84 (1.07-3.14), respectively]. Carriers of the A allele showed reduced expression of CD209 and TGF-β1 in leprosy lesions in comparison with individuals with GG genotype, in addition to a higher response to the Mitsuda test.

**CONCLUSION:**

These data suggest that rs735240 influences the immune response against *M. leprae* and clinical presentation of leprosy.

Leprosy is considered a public health problem in Brazil, which registered around 28,000 new cases among a total of 202,185 cases detected worldwide in 2019.^1^ Although the introduction of multidrug therapy has reduced the prevalence of the disease, epidemiological indices still show active transmission of *Mycobacterium leprae*.^2^ Besides, leprosy presents important morbidity associated with peripheral nerve damage.^3^

Following the initial host-pathogen interaction, elimination of the bacillus or clinical manifestation of leprosy in several forms may occur.^4^ The diversity of the possible outcomes reflects the host immune response.^5^ ^,^ ^6^ Two polar and stable forms of leprosy are known: the tuberculoid form (TT), considered restrictive, which presents as a localised disease with few lesions and rare bacilli due to the intense cellular immune response, and the lepromatous form (LL) with disseminated lesions and a large number of bacilli that result from an inefficient immune response characterised by the inhibition of macrophage antimicrobial activity, high levels of antibodies, and activation of regulatory T cells.^7^ Three intermediate and unstable forms exist between these poles: borderline tuberculoid (BT), borderline borderline (BB), and borderline lepromatous (BL), among which the cellular response gradually decreases from the tuberculoid to the lepromatous pole.^5^ ^,^ ^8^ For treatment purposes, leprosy patients are classified as paucibacillary (PB) or multibacillary (MB), according to the number of lesions or the bacillary index, when available.^9^

*Mycobacterium leprae* has a compact genome^10^ and exhibits low genetic diversity.^11^ Thus, the complexity of clinical and biological phenotypes in leprosy points to the major role of the host genetic background in determining the course of the disease.^12^ ^,^ ^13^ This fact has been evidenced by classical and molecular genetic studies demonstrating that disease susceptibility is influenced by genes that also regulate the progression of leprosy.^14^ Some genes, such as NOD2, CCDC122-LACC1, TNF, TLR1, IFNG, and IL10, have already been associated with leprosy susceptibility after being consistently replicated in different populations.^15^ ^,^ ^16^ ^,^ ^17^ ^,^ ^18^ ^,^ ^19^ However, genes may also be involved in the clinical manifestations of leprosy.^20^

The *CD209* gene encodes DC-SIGN (CD209), a C-type lectin receptor expressed on the surface of dendritic cells (DCs), that is involved in mycobacterial recognition,^21^ ^,^ ^22^ promoting antigen internalisation, and presentation.^23^ DC-SIGN binding by mycobacteria impairs DC maturation and promotes pathogen persistence.^24^ Previous studies have shown that polymorphisms in the promoter region of *CD209* are associated with susceptibility to several infectious diseases^25^ ^,^ ^26^ ^,^ ^27^ including tuberculosis.^28^ ^,^ ^29^ In addition, other studies associated these polymorphisms with the severity of tuberculosis,^30^ hepatitis C,^31^ severe acute respiratory syndrome^32^ and dengue fever.^33^ ^,^ ^34^

Considering that the recognition of *M. leprae* involves DC-SIGN, but polymorphisms in this gene were not associated with leprosy susceptibility,^22^ we investigated the association of single nucleotide variants (SNVs) in the promoter region of the *CD209* gene with clinical forms of leprosy in the Brazilian population. In addition, we investigated the functional effects of these polymorphisms on the response to *M. leprae*.

## SUBJECTS AND METHODS

*Participants and study design* - The association of rs2287886, rs4804803, rs735239, and rs735240 SNVs in the *CD209* gene promoter region with clinical forms of leprosy was tested in a Middle Eastern Brazilian population of 406 leprosy patients from Rondonópolis, Mato Grosso state. This is an endemic area in Brazil, with a prevalence of 15.52 leprosy cases per 10,000 inhabitants in 2018.^35^ Leprosy diagnosis was confirmed by clinical and laboratory tests performed at the outpatient service in Rondonópolis and Lauro de Souza Lima Institute, respectively. The general characteristics of the participants are presented in Table I. The classification of patients with leprosy was based on the Ridley and Jopling (R&J) criteria,^5^ which considers clinical, histopathological, immunological, and bacilloscopic characteristics. Bacilloscopic evaluation was performed on the histological sections of the skin samples using the Fite-Faraco technique and corresponded to the bacilloscopic index (BI, 0-6+) associated with the evaluation of the presence of bacilli in different skin tissues (mainly in granulomas). This information (clinical, histopathological, immunological, and bacilloscopic characteristics) allows for classification in the R&J spectrum (TT, BT, BB, BL, and LL). For genetic and functional analysis, samples with BI “0” or 1+ were considered paucibacillary, which represented patients I, TT, and some BT (PB, n = 96). Samples with BI ≥ 2+ were considered multibacillary and represented some BT patients and all BB, BL, and LL patients (MB, n = 310). This modified version of the operational classification recommended by the WHO^36^ ^,^ ^37^ was adopted since it reflects the ability of patients to contain bacillary multiplication.^38^ ^,^ ^39^ To better characterise the clinical condition of leprosy patients, other clinical and laboratory data from the patients, such as disability grade, bacterial index, antibody response against PGL-I antigen from *M. leprae*, and skin response in the Mitsuda reaction were obtained from medical reports.

**TABLE I:** Characteristics of leprosy patients from Rondonópolis - Mato Grosso state enrolled in the genetic study (n = 406)

Characteristics	Categories	MB (n = 310)	PB (n = 96)
Age (mean ± SD)		42.9 ± 16.1	39.1 ± 16.1
Sex (n/%)	Male Female	201 (64.8%) 109 (35.2%)	45 (46.9%) 51 (53.1%)
Ridley and Jopling classification (n/%)	LL BL BB BT TT I	21 (6.8%) 63 (20.3%) 79 (25.5%) 147 (47.4%) --- ---	--- --- --- 9 (9.4%) 60 (62.5%) 27 (28.1%)

SD: standard deviation; LL: lepromatous-lepromatous leprosy; BL: borderline-lepromatous leprosy; BB: borderline-borderline leprosy; BT: borderline-tuberculoid leprosy; TT: tuberculoid-tuberculoid leprosy; I: indeterminate leprosy.

For ethnicity control, molecular ancestry was defined using 46 ancestry-informative indels, as previously described.^40^ ^,^ ^41^ The estimates of individual ancestry (European, African, and Native American) were analysed using the ADMIXTURE software.^42^

*SNVs selection* - The candidate SNVs rs2287886 (-139A>G), rs4804803 (-336A>G), rs735239 (-871A>G), and rs735240 (-939G>A) were chosen considering the previous association of the *CD209* promoter region with the severity of infectious diseases.^31^ ^,^ ^43^ ^,^ ^44^ Moreover, the *CD209* promoter region has different binding sites for transcription factors, such as AP-1, Sp-1, Ets-1, and NF-kb, and the proximity of these sites has the potential to affect the transcriptional levels.^45^

*DNA extraction and SNVs genotyping* - For genetic analysis, genomic DNA was extracted from the peripheral blood leukocytes using the *salting-out* method.^46^ For the functional analysis, DNA was obtained from skin lesion biopsies embedded in paraffin. These samples were processed using the Qiagen DNA FFPE Tissue Kit (Qiagen, Hilden, Germany). Genotyping was performed by allelic discrimination using fluorogenic probes (TaqMan assay numbers: C_1999341_10 (rs735240), C_1999340_10 (rs4804803), C_989421_10 (rs735239), and C_11515683_1 (rs2287886) using Viia 7 equipment (Applied Biosystems, Foster City, CA, USA), according to the manufacturer’s instructions.

*Functional evaluation* - To evaluate the functional effects of the rs735240 SNV on the immune response against *M. leprae,* we analysed the expression levels of DC-SIGN, cytokines, and other accessory molecules using data from two previous and independent studies from our group.^47^ ^,^ ^48^ First, we examined the mRNA expression levels in 28 leprosy patients [Supplementary data (Table I)] according to the presence or absence of allele A of rs735240 using the dataset GSE74481.^47^ In brief, biopsies from leprosy skin lesions were collected and stored in RNAlater solution (Ambion). RNA extraction was performed using a Precellys24 apparatus (Bertin Technologies), and RNA integrity was evaluated using a 2100 Bioanalyzer electrophoresis apparatus (GE Healthcare Bio-Sciences). Total RNA was reverse transcribed and amplified, and gene expression was evaluated using the 8X60K cDNA microarray G4858A platform (GE Healthcare Bio-Sciences) containing 60,000 probes representing the entire human genome. The microarray data were analysed using Gene Spring GX version 12.1 software (Agilent Technologies).

We also explored the expression of DC-SIGN, cytokines, and other surface markers by monocyte-derived DCs from leprosy patients primed with *M. leprae* antigen^48^ [Supplementary data (Table II)], taking into account the genotype of rs735240. In this study, monocytes from leprosy patients were purified from peripheral blood and treated with GM-CSF and IL-4 to differentiate into DCs that were stimulated by *M. leprae* sonicated antigen or standard maturation cocktail (IL-1β, IL-6, tumor necrosis factor, and prostaglandin E2), or remained unstimulated. After 48 h, the supernatant was collected and stored at -80**º**C for cytokine measurement using commercial enzyme-linked immunosorbent assay (ELISA) kits (R&D Systems, USA). DCs were harvested for analysis of surface marker expression by flow cytometry, as previously described.^48^

Furthermore, we analysed the influence of this variant on clinical and laboratory parameters in patients with leprosy recruited for the genetic study.

*Data analysis* - The genotypes, alleles, and carrier frequencies were compared using a univariate logistic regression model. These analyses were also performed with adjustments for covariates of sex and molecular ancestry. The last variable was used for continuous adjustment because there is no consensus regarding the use of these continuous variables to classify ethnicity as a categorical variable. Statistical software R version 2.5.1 for Windows and the Genetics package were used. A p-value < 0.05 was adopted as the cut-off for statistical significance.

For functional analyses, data were grouped into carriers and non-carriers of allele A from rs735240 SNV (AA+AG *vs* GG). Statistical analysis was performed using the non-parametric Mann-Whitney test, and significance was set at p-value < 0.05. All analyses were performed using the GraphPad Prism software version 7.04 (GraphPad Software Inc., La Jolla, CA, USA, 2017).

*Ethics* - This study was approved by the Research Ethics Committee of the Lauro de Souza Lima Institute (CAAE 68433217.0.0000.5475), and all protocols were carried out in accordance with the Helsinki Declaration of 1975, as revised in 1983, as well as Brazilian law concerning ethics in biological research.

## RESULTS

*rs735240 SNV was associated with the clinical form of leprosy* - Genotyping was successful in more than 90% of the samples for all four SNVs studied. The heterozygous GA genotype of rs735240 SNV was significantly associated with paucibacillary leprosy (OR 2.02; CI 1.10-3-73; p = 0.0240). In addition, an association between A allele carriers and paucibacillary leprosy was noted (OR 1.85; CI 1.04-3.31; p = 0.0350) (Table II).

The rs2287886, rs4804803, and rs735239 SNVs were not associated with clinical forms of leprosy, as summarised in Table II.

**TABLE II:** Alelle, genotype and carrier frequencies of the rs735240, rs4804803, rs735239 and rs2287886, polymorphisms in *CD209* promoter region of leprosy patients. Logistic regression data for association with clinical forms of leprosy at the sample from Rondonópolis, MT, Brazil

Polymorphisms	Categories	PB	MB	OR (95% CI) p-value	OR (95% CI) p-value^*a*^
rs735240 -939G>A	G	94 (0.52)	318 (0.57)	*	*
	A	88 (0.48)	240 (0.43)	1.2 (0.8-2.0) 0.37	1.31 (0.8-2.2) 0.30
	GG	22 (0.24)	103 (0.37)	*	*
	GA	50 (0.55)	112 (0.40)	2.1 (1.2-3.7) 0.01	2.0 (1.1-3.7) 0.02
	AA	19 (0.21)	64 (0.23)	1.4 (0.7-2.8) 0.35	1.6 (0.8-3.2) 0.22
	A carrier	69 (0.66)	176 (0.63)	1.8 (1.1-3.1) 0.03	1.8 (1.0-3.3) 0.04
	Total	91	279		
rs4804803 -336A>G	A	146 (0.77)	445 (0.73)	*	*
	G	44 (0.23)	165 (0.27)	0.8 (0.5-1.4) 0,45	0.8 (0.4-1.4) 0.47
	AA	53 (0.56)	165 (0.54)	*	*
	AG	40 (0.42)	115 (0.38)	1.1 (0.7-1.7) 0.74	1.1 (0.6-1.8) 0.74
	GG	2 (0.02)	25 (0.08)	0.2 (0.1-1.1) 0.06	0.2 (0.1-1.1) 0.07
	G carrier	42 (0.44)	140 (0.46)	0.9 (0.6-1.5) 0.77	0.9 (0.6-1.6) 0.80
	Total	95	305		
rs735239 -871A>G	A	121 (0.67)	377 (0.68)	*	*
	G	59 (0.33)	177 (0.32)	1.0 (0.6-1.7) 0.88	1.1 (0.6-1.9) 0.69
	AA	39 (0.43)	133 (0.48)	*	*
	AG	43 (0.48)	111 (0.40)	1.3 (0.8-2.2) 0.28	1.4 (0.8-2.4) 0.21
	GG	8 (0.09)	33 (0.12)	0.8 (0.3-1.9) 0.66	1.0 (0.4-2.3) 0.95
	G carrier	51 (0.57)	144 (0.52)	1.2 (0.7-1.9) 0.44	1.3 (0.8-2.2) 0.31
	Total	90	277		
rs2287886 -139A>G	G	130 (0.69)	406 (0.70)	*	*
	A	58 (0.31)	170 (0.30)	1.1 (0.6-1.8) 0.81	1.00 (0.6-1.7) 0.97
	GG	43 (0.46)	147 (0.51)	*	*
	GA	44 (0.47)	112 (0.39)	1.3 (0.8-2.2) 0.23	1.2 (0.7-2.0) 0.48
	AA	7 (0.07)	29 (0.10)	0.8 (0.3-2.0) 0.67	0.7 (0.3-1.9) 0.54
				
	A carrier	51 (0.54)	141 (0.49)	1.2 (0.8-2.0) 0.37	1.1 (0.7-1.8) 0.68
	Total	94	288		

*a*: adjusted regression values using sex and molecular ancestry covariates. CI: confidence interval; MB: multibacillary leprosy patients; OR: odds ratio; PB: paucibacillary leprosy patients.

*rs735240 SNV presented functional effects in the immune response against M. leprae* - Considering the association of rs735240 with PB leprosy as well as the relevance of DC-SIGN in the recognition of mycobacteria by DCs, we analysed the influence of this variant on the immune response against *M. leprae* by evaluating the expression of *CD209* and other molecules involved in DC activation. Allele A carriers showed lower expression of DC-SIGN in both leprosy skin lesions and monocyte-derived DCs stimulated with *M. leprae,* with marginal significance (Figure A-B) in comparison with non-carriers (GG genotype). In addition, the levels of TGF-β1, a cytokine induced after DC-SIGN activation, were smaller in allele A carriers under the same conditions (Figure C-D). The expression levels of *CD40*, *ITGAX* (CD11c), *CCL17*, *CD83*, *CIITA*, *IL1B*, *IL6*, *ISG15*, *OAS1*, *RNaseL*, *TLR3*, and *TNF* genes in skin lesions, as well as CD11c, CD40, CD80, CD86, HLA-DR, ICAM-1, IL-10, IL-12-23p40, IL-12p70, and TNF, which are expressed after DC activation, were also evaluated according to the rs735240 SNV, but no significant differences were found (data not shown). Although the findings for the expression levels of CD209 and TGF-β1 in leprosy lesions and DC cultures are in accordance, the data obtained *in vitro* included few patients and only two individuals with the GG genotype for rs735240, therefore requiring further confirmation.

Furthermore, we also observed that the rs735240 allele A has some influence on the response to the Mitsuda test in leprosy patients. The Mitsuda test is a skin reaction based on a delayed cell-mediated immune response against mycobacteria; it is used for the prognostic and clinical classification of leprosy.^49^ We observed that allele A carriers exhibited a higher Mitsuda response than non-carriers (Figure E). The response to the Mitsuda test observed in AA allele carriers was higher than that observed in GG individuals for both PB (5,7 mm x 4,8 mm) and MB patients (4,2 mm and 3,1 mm). Disability grade, bacterial index, and antibody response against the PGL-I antigen from *M. leprae* showed no difference related to the rs735240 SNV (data not shown).

**Figure:**
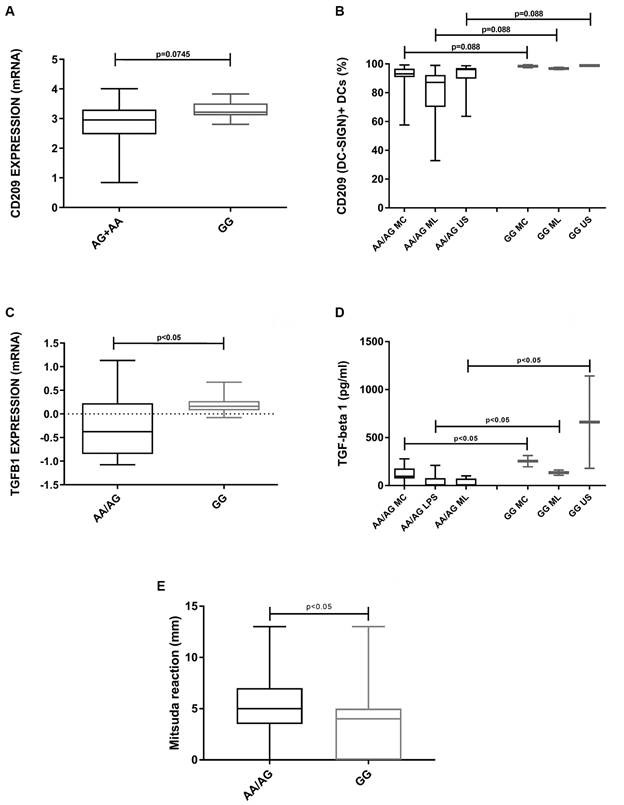
Functional effects of rs735240 SNV at *CD209* gene in the immune response against *Mycobacterium leprae.* The expression of CD209 (DC-SIGN) and TGF-β1 according to the presence or absence of the allele A was evaluated in leprosy skin lesions (A and C) and monocyte-derived dendritic cells stimulated with *M. leprae* antigens (B and D). Besides, the response to the Mitsuda test in leprosy patients was analyzed in allele A carriers (n = 98) and non-carriers (n = 73). MC: DCs stimulated for 48 h with a specific maturation cocktail IL-1β (25 ng/mL), IL-6 (1,000 U/mL), tumor necrosis factor (50 ng/mL) and prostaglandin E2 (10^-6^ M); ML: DCs stimulated with sonicated antigen of *M. leprae* (10 μg/mL); US: unstimulated immature DCs. Mann-Whitney test was used to statistical analysis.

## DISCUSSION

Although polymorphisms located in the promoter region of *CD209* are widely associated with the severity of various infectious diseases,^31^ ^,^ ^32^ ^,^ ^44^ ^,^ ^50^ ^,^ ^51^ including tuberculosis,^30^ such association has not yet been reported in leprosy. In view of the relevance of DC-SIGN in the recognition of antigens and activation of the immune response, and considering that the variations in genes that potentially influence the course of infections are largely population-dependent^52^ we chose to investigate the association of candidate variants in the CD209 promoter region with the clinical manifestation of leprosy in a Brazilian population. We observed an association between the A allele and AG genotype of SNV rs735240 (-939G>A) with paucibacillary leprosy, a localised form of the disease. No other studies in the Brazilian population have investigated the association between *CD209* SNVs and the clinical forms of leprosy. Worldwide, only two studies have investigated this association: one in a Japanese population evaluating the -336 SNV (rs4804803)^53^ and the other investigating seven SNVs in a Pakistani population;^22^ both being non-significant associations. However, it is important to mention that these studies were performed with a limited casuistic comparing 60 and 109 MB to 33 and 85 PB patients in Japan and Pakistan, respectively, while our study included 310 MB and 96 PB patients, rendering robust the data presented here.

Silva et al.^54^ observed that the rs735240 SNV was associated with extrapulmonary tuberculosis susceptibility, rs2287886 showed a significant association with pulmonary tuberculosis, and rs4804803 and rs735239 provided protection against the disease, confirming the association of the promoter region in CD209 with mycobacterial diseases. rs4804803 also contributes towards genetic susceptibility to ulcerative colitis^55^ in which the colonisation of the intestinal tract by mycobacteria has been described.

Considering other infectious diseases, the A allele of rs735240 is associated with the development of fungal keratitis in the Han Chinese population.^56^ In addition, a higher incidence of cytomegalovirus (CMV) infection among kidney transplant recipients not receiving prophylaxis was associated with the GG genotype of rs735240,^57^ and CMV reactivation after allogeneic stem cell transplantation was associated with the G allele of rs735240.^58^

Furthermore, the A allele of rs735240 was associated with protection against Kawasaki disease, a systemic vasculitis of unknown etiology,^59^ indicating that this SNV can modulate the immune response in different scenarios. In fact, the promoter region of the CD209 gene contains binding sites for the transcription factors AP-1, Sp-1, Ets-1, and NF-κB that participate in the activation of the immune system.^45^

Our results showed lower expression of *CD209* mRNA related to the A allele of rs735240 SNV in skin lesions from leprosy patients, as well as a tendency to decrease DC-SIGN expression levels in monocyte-derived DCs, even if this needs to be considered with caution because of the low number of patients evaluated *in vitro*. *M. leprae* is recognised via DC-SIGN,^22^ ^,^ ^60^ the activation of which triggers pathways associated with IL-10 secretion and results in an immunosuppressive response that contributes to pathogen persistence.^24^ Thus, the lower expression levels of DC-SIGN in A allele carriers could imply a low production of IL-10, concurring with the development of an efficient cell-mediated immune response observed in PB leprosy patients. Similarly, Li et al.^61^ proposed that the AA genotype of rs735240 can protect against nasopharyngeal carcinoma by reducing DC-SIGN expression and consequently decreasing the infection of DCs and nasopharyngeal epithelial cells by the Epstein-Barr virus. In addition, Vannberg et al. observed that the GG genotype of the rs4804803 SNV is involved in the downregulation of CD209 mRNA expression, which protects against a severe form of tuberculosis characterised by pulmonary cavitation.^30^

Our study also showed that carriers of the A allele presented lower TGB-β1 expression in skin lesions and reduced levels of this cytokine in cultures of monocyte-derived DCs stimulated with *M. leprae* antigens. This cytokine is involved in the development and maintenance of suppressor and regulatory cells in the immune response.^62^ In leprosy, the TGF-β expression levels were higher in the skin lesions from LL and BL patients with a high bacilloscopic index than in paucibacillary patients,^8^ ^,^ ^63^ as well as in PBMCs of these individuals stimulated with *M. leprae* antigen.^64^ Lower production of TGB-β1 by A allele carriers may avoid bacillary persistence and multiplication, favoring the control of infection and the development of PB leprosy, the most restrictive form of the disease.

Another important finding in our study is the association of the A allele of rs735240 with a greater response to the Mitsuda test, an intradermic reaction based on the injection of heat-killed *M. leprae.* The test is considered positive when a granulomatous response with a size >5 mm occurs, which reflects a cell-mediated immune response that is able to control mycobacterial infection. The reaction is usually positive in patients with PB with localised diseases. Although, based on our data, it is not possible to determine the mechanisms involved in this association, it is reasonable to suggest that rs735240 could influence the polarisation of the immune response and favor the development of PB leprosy.

A limitation of our study is that both genetic and functional analyses involved multiple tests and would require statistical corrections. However, we did not apply these extremely conservative corrections methods as they could over-correct the data. The non-correction decision for genetic study considered the strong linkage disequilibrium between the variants and that some authors discuss the marker interdependence as a parameter for adjusting correction methods.^65^ Furthermore, the functional findings were replicated in two distinct sets of data and agree with the observed genetic association, reinforcing that our findings were not by chance.

Although the influence of genetic background on leprosy development has been extensively investigated, few studies have evaluated the role of genes in the progression towards different clinical forms. This is especially important for identifying markers of disseminated disease associated with leprosy transmission. In summary, our results indicate, for the first time, an association between rs735240 and the *CD209* gene and leprosy immunological dichotomy, as has been observed in other infectious diseases. In addition, this variant had a functional influence on the immune response against *M. leprae*.
