# Supplementary material for: Association of CD209 (DC-SIGN) rs735240 SNV with paucibacillary leprosy in the Brazilian population and its functional effects
Source: Mem Inst Oswaldo Cruz. 2022 Jun 10;117:e220014. doi: 10.1590/0074-02760220014 (PMC9190517; doi:10.1590/0074-02760220014)
Supplement: Supplementary file 1 [file 1678-8060-mioc-117-e220014-s.pdf]

TABLE I
Characteristics of leprosy patients enrolled in the study of mRNA expression in skin lesions (n = 28)

		Genotype for rs735240 SNP	
Characteristics	Categories	AA/AG (n = 19)	GG (n = 9)
Age (mean ± SD)		41.8 ± 10.6	41.2 ± 13.4
Sex (n/%)	Male	14 (73.7%)	4 (44.4%)
	Female	5 (26.3%)	5 (55.6%)
Clinical form (n/%)	MB	9 (47.4%)	5 (55.6%)
	PB	10 (52.6%)	4 (44.4%)

MB: multibacillary; PB: paucibacillary; SD: standard deviation.

TABLE II
Characteristics of leprosy patients enrolled in the study of DC-SIGN expression and production of cytokines in cultures of monocyte-derived dendritic cells stimulated with *Mycobacterium leprae* antigens (n = 14)

		Genotype for rs735240 SNP	
Characteristics	Categories	AA/AG (n = 12)	GG (n = 2)
Age (mean ± SD)		45.8 ± 15.7	42.5 ± 30.4
Sex (n/%)	Male	9 (75.0%)	2 (100.0%)
	Female	3 (25.0%)	0 (0.0%)
Clinical form (n/%)	MB	8 (66.7%)	1 (50.0%)
	PB	4 (33.3%)	1 (50.0%)

MB: multibacillary; PB: paucibacillary; SD: standard deviation.
